# Cytoreductive surgery plus hyperthermic intraoperative peritoneal chemotherapy for people with peritoneal metastases from colorectal, ovarian or gastric origin: A systematic review of randomized controlled trials

**DOI:** 10.1002/wjs.12186

**Published:** 2024-04-24

**Authors:** Kurinchi Gurusamy, Jeffrey Leung, Claire Vale, Danielle Roberts, Audrey Linden, Xiao Wei Tan, Priyal Taribagil, Sonam Patel, Elena Pizzo, Brian Davidson, Mark Saunders, Omer Aziz, Sarah T. O’Dwyer

**Affiliations:** 1 University College London, London, UK; 2 The Colorectal and Peritoneal Oncology Centre, Christie NHS Foundation Trust, London, UK; 3 Division of Cancer Studies, University of Manchester, London, UK

**Keywords:** cost-effectiveness analysis, cost-utility analysis, evidence-based medicine, hyperthermic intraoperative peritoneal chemotherapy, meta-analysis, peritoneal metastases, probabilistic sensitivity analysis, systematic review, value of information analysis

## Abstract

**Background:**

There is uncertainty in the relative benefits and harms of hyperthermic intraoperative peritoneal chemotherapy (HIPEC) when added to cytoreductive surgery (CRS) +/− systemic chemotherapy or systemic chemotherapy alone in people with peritoneal metastases from colorectal, gastric, or ovarian cancers.

**Methods:**

We searched randomized controlled trials (RCTs) in the medical literature until April 14, 2022 and applied methods used for high-quality systematic reviews.

**Findings:**

We included a total of eight RCTs (seven RCTs included in quantitative analysis as one RCT did not provide data in an analyzable format). All comparisons other than ovarian cancer contained only one trial. For gastric cancer, there is high uncertainty about the effect of CRS + HIPEC + systemic chemotherapy. For stage III or greater epithelial ovarian cancer undergoing interval cytoreductive surgery, CRS + HIPEC + systemic chemotherapy probably decreases all-cause mortality compared to CRS + systemic chemotherapy. For colorectal cancer, CRS + HIPEC + systemic chemotherapy probably results in little to no difference in all-cause mortality and may increase the serious adverse events proportions compared to CRS +/− systemic chemotherapy, but probably decreases all-cause mortality compared to fluorouracil-based systemic chemotherapy alone.

**Interpretation:**

The role of CRS + HIPEC in gastric peritoneal metastases is uncertain. CRS + HIPEC should be standard of care in women with stage III or greater epithelial ovarian cancer undergoing interval CRS. CRS + systemic chemotherapy should be standard of care for people with colorectal peritoneal metastases, with HIPEC given only as part of a RCT focusing on subgroups and regimes.

**PROSPERO Registration:**

CRD42019130504.

## 1 Background and Rationale

### 1.1 What is the problem being addressed?

Approximately seven million people worldwide and 160,000 people in the UK develop colorectal, ovarian, or gastric cancer each year,^1^ of whom 8%–50% develop peritoneal metastases. The peritoneum is one of the commonest sites of metastases from these cancers.^2–8^ In general, people with peritoneal metastases have poorer prognosis than those with other sites of metastases (liver or lung),^9^ with median reported survival ranging from 6 to 24 months.^10,45,52^

### 1.2 Treatment of peritoneal metastases from colorectal, ovarian, or gastric cancer

The current standard of care of people with peritoneal metastases from these cancers is systemic chemotherapy either alone or in combination with cytoreductive surgery (CRS) or palliative surgery.^4,7,11,12,45,52^ The addition of hyperthermic intraoperative peritoneal chemotherapy (HIPEC) to CRS + systemic chemotherapy is an option, and was commissioned for colorectal peritoneal metastases by NHS England in 2013. The main principle of CRS + HIPEC is to remove all visible (macroscopic) peritoneal metastases by surgical resection (CRS) followed by HIPEC to treat any remaining microscopic peritoneal metastases.^13^ HIPEC involves peritoneal circulation of chemotherapy drugs (usually mitomycin C, oxaliplatin with 5 fluorouracil, or cisplatin)^14^ heated to temperatures of 42°C, which might potentiate the chemotherapy drugs.^15^

### 1.3 Why is this research important to patients and health and care services?

Although CRS + HIPEC has the potential to improve the survival and health-related quality of life (HRQoL) in people with peritoneal metastases,^11,16,17^ there have been concerns raised about its safety. Whilst some reports have shown a 30-day mortality after CRS + HIPEC of 1%–3%,^2^ and a major complication rate of 32%,^2,18^ data from high volume centers has shown that major complication rates are around 10%–15% and a 90-day mortality of 1%.^19^ The average costs of CRS + HIPEC per patient varies from about 20,000–80,000 USD.^20–26^ Because of these reasons, this research is important to address the significant uncertainty about the benefits of an intervention that carries potential risk of harm to patients and major costs to the NHS.

### 1.4 Review of existing evidence

Prior to starting this research, 16 systematic reviews of comparative studies had been undertaken, comparing CRS + HIPEC to other treatment modalities in peritoneal metastases from colorectal, ovarian, or gastric cancer.^2,14,16,27–39^ 10 of these included at least one randomized controlled trial (RCT), but the conclusions were largely based on non-randomized studies.^2,14,16,27,29–31,33,38,39^ Although most of these systematic reviews concluded that CRS + HIPEC can improve survival in people with peritoneal metastases, all the systematic reviews had limitations and deficiencies. Firstly, all were at high risk of bias according to the ROBIS (Risk Of Bias In Systematic reviews) tool^40^ with concerns about bias across all domains. Secondly, the systematic reviews included only a single RCT^45^ and/or based their evidence predominantly on non-randomized studies, without any adjustment for baseline differences in disease-related or patient-related prognostic characteristics.^2,14,16,27,29–31,33,38,39^ Finally, meta-analyses could only include a small proportion of the results from the studies because of the way these results had been reported (e.g., proportion survived vs. median survival).^14,16,27,33,35^

## 2 Aims and Objectives

The overarching aim of this project is to answer whether CRS + HIPEC + systemic chemotherapy improves survival and/or quality of life compared to CRS +/− systemic chemotherapy or systemic chemotherapy alone in people with peritoneal metastases (from colorectal, gastric, or ovarian cancers) who can withstand major surgery and is it cost-effective in the NHS setting by a systematic review and cost-effectiveness analysis (CEA). In this report, we have provided the results of the systematic review. We have provided the results of the CEA in the full report from NIHR.

## 3 Methods

We performed a systematic review of literature by searching MEDLINE, EMBASE, Cochrane library, Science Citation Index, Conference Proceedings Citation Index as well as trial registers until April 14, 2022. The search strategies are available in Appendix A. We followed the standard guidance for performing a high-quality systematic review and meta-analysis. We included only RCTs and assessed the risk of bias using the Risk of Bias version 2·0 (ROB 2·0).^41^ We calculated the hazard ratio (HR), risk ratio (RR), rate ratio, or mean difference (MD) with 95% confidence intervals (95% CI) as appropriate. When applicable, we performed meta-analysis using the random-effects model using Review Manager 5·4. We used GRADE guidance to assess the certainty of evidence and determine the strength of recommendations.^42^

For detailed methods of performing the systematic review, please see our published protocol^43^ and Supporting Information S1 (accepted for publication in NIHR Journals).

## 4 Role of Funding Source

The funder sought independent peer review before funding and approved the protocol. All protocol revisions were approved by the funder.

## 5 Results

**Figure 1:**
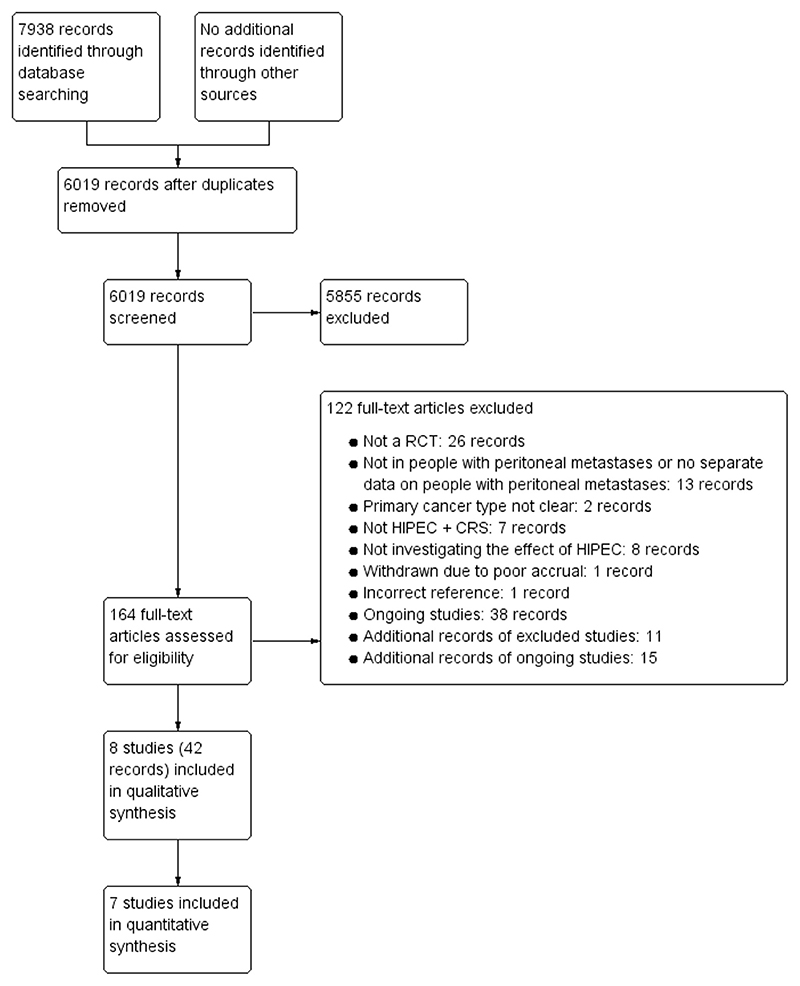
Study flow diagram. Abbreviations: CRS, cytoreductive surgery; HIPEC, hyperthermic intraperitoneal chemotherapy; RCT, randomized control trial.

The systematic review included a total of eight RCTs. A total of 955 participants in seven RCTs were included in quantitative analysis (Table 1). Further details of HIPEC and systemic chemotherapy in these studies are summarized in Appendix B (Tables B1 and B2). All comparisons other than that for ovarian cancer contained only one trial. We excluded 5855 clearly irrelevant records through reading titles and abstracts. We excluded 58 records: the reasons for exclusion are available in our full report. We identified 38 records of ongoing trials (available from our full report). Additional reports of included, excluded, and ongoing studies (60 records) are listed in our full report. The reference flow is shown in Figure 1. The risk of bias in the different domains for mortality are shown in Table 2. The certainty of evidence and the reasons for downgrading the evidence are available in Table 3. Most of the evidence related to all-cause mortality was of moderate certainty.

### 5.1 Gastric peritoneal metastases

#### 5.1.1 CRS + HIPEC + systemic chemotherapy versus CRS + systemic chemotherapy

One trial (68 participants) provided data in analyzable format,^44^ while another trial did not provide data in analyzable format but provided a narrative statement about all-cause mortality.^45^ For gastric cancer, there is high uncertainty about the effect of CRS + HIPEC + systemic chemotherapy versus CRS + systemic chemotherapy on all-cause mortality and serious adverse events (effect estimates not presented because of very low certainty evidence).

#### 5.1.2 CRS + HIPEC + systemic chemotherapy versus systemic chemotherapy

One trial (17 participants) was included in the analysis.^46^ CRS + HIPEC + systemic chemotherapy probably decreases all-cause mortality compared to systemic chemotherapy (effect estimates not presented because of high degree of uncertainty in evidence).

### 5.2 Ovarian cancer

#### 5.2.1 CRS + HIPEC + systemic chemotherapy versus CRS + systemic chemotherapy (stage III or above requiring interval CRS)

**Figure 2:**
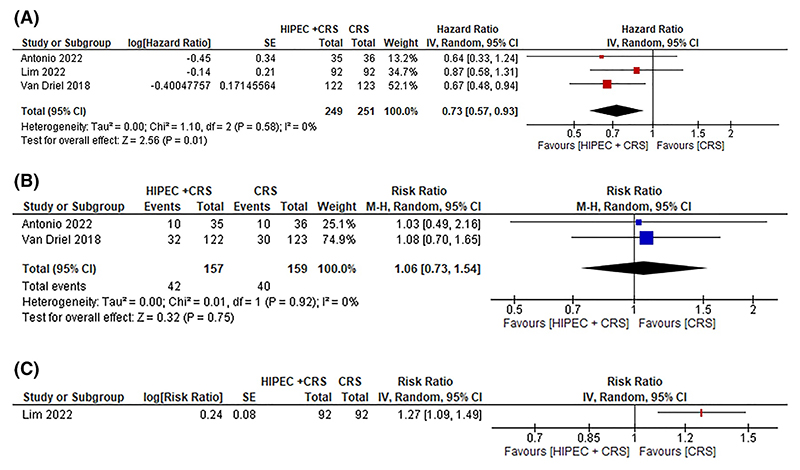
Ovarian cancer: CRS + HIPEC + systemic chemotherapy versus CRS + systemic chemotherapy. (A) All-cause mortality. Abbreviations: HIPEC, hyperthermic intraperitoneal chemotherapy; CRS, cytoreductive surgery; SE, standard error; 95% CI, 95% confidence interval. The figure shows that CRS + HIPEC + systemic chemotherapy probably results in lower mortality and disease progression than CRS + systemic chemotherapy. (B) Serious adverse events (Proportion). Abbreviations: HIPEC, hyperthermic intraperitoneal chemotherapy; CRS, cytoreductive surgery; 95% CI, 95% confidence interval. The figure also shows that there may be little or no differences in the proportion of participants who developed serious adverse events between CRS + HIPEC + systemic chemotherapy and CRS + systemic chemotherapy. (C) Serious adverse events (Number per participant). Abbreviations: HIPEC, hyperthermic intraperitoneal chemotherapy; CRS, cytoreductive surgery; SE, standard error; 95% CI, 95% confidence interval. The figure also shows that the number of serious adverse events were probably higher in CRS + HIPEC + systemic chemotherapy compared to CRS + systemic chemotherapy. [Colour figure can be viewed at wileyonlinelibrary.com]

Three trials (500 participants) compared CRS + HIPEC + systemic chemotherapy versus CRS + systemic chemotherapy.^47–49^ For stage III or greater ovarian cancer requiring interval cytoreductive surgery, CRS + HIPEC + systemic chemotherapy probably decreases all-cause mortality compared to CRS + systemic chemotherapy (46·3% in CRS + HIPEC + systemic chemotherapy vs. 57·4% in CRS + systemic chemotherapy;median follow-up 32–70 months; HR 0·73; 95% CI 0·57 to 0·93; 3 trials; 500 participants; moderate certainty evidence) (Figure 2A). It may result in little to no difference in HRQoL (MD 4·85; 95% CI -7·74 to 17·44; 1 trial; 71 participants; moderate certainty evidence) or number of people who developed serious adverse events compared to CRS + systemic chemotherapy (26·7% in CRS + HIPEC + systemic chemotherapy vs. 25·2% in CRS + systemic chemotherapy; RR 1·06; 95% CI 0·73 to 1·54; 2 trials; 316 participants; moderate certainty evidence) (Figure 2B), although it probably increases the number of serious adverse events per participant compared to CRS + systemic chemotherapy (41·4 events per 100 participants in CRS + HIPEC + systemic chemotherapy vs. 32·6 events per 100 participants in CRS + systemic chemotherapy; rate ratio 1·27; 95% CI 1·09 to 1·49; 1 trial; 184 participants; moderate certainty evidence) (Figure 2C).

### 5.3 Colorectal peritoneal metastases

#### 5.3.1 CRS + HIPEC + systemic chemotherapy versus CRS + systemic chemotherapy

One trial (265 participants) was included in the analysis.^50^ For colorectal cancer, CRS + HIPEC to systemic chemotherapy probably results in little to no difference in all-cause mortality compared to CRS and systemic chemotherapy without HIPEC (60·6% in CRS + HIPEC + systemic chemotherapy vs. 60·6% in CRS + systemic chemotherapy; median follow-up 64 months; HR 1·00; 95% CI 0·63 to 1·58; 1 trial; 265 participants; moderate certainty evidence). The addition of HIPEC may increase the number of people who develop serious adverse events compared to CRS +/− systemic chemotherapy (25·6% in CRS + HIPEC + systemic chemotherapy vs. 15·2% in CRS + systemic chemotherapy; RR 1·69; 95% CI 1·03 to 2·77; 1 trial; 265 participants; low certainty evidence).

#### 5.3.2 CRS + HIPEC + systemic chemotherapy versus systemic chemotherapy

One trial (105 participants) was included in the analysis.^51^ CRS + HIPEC + systemic chemotherapy probably decreases all-cause mortality compared to fluorouracil-based systemic chemotherapy alone (40·8% in CRS + HIPEC + systemic chemotherapy vs. 60·8% in systemic chemotherapy alone; median follow-up 22 months; HR 0·55; 95% CI 0·32 to 0·95; 1 trial; 105 participants; moderate certainty evidence).

### 5.4 Subgroup and sensitivity analysis

We did not perform any of the planned subgroup analysis because of sparse data. The sensitivity analyses did not alter the interpretation of data or conclusions.

### 5.5 Reporting bias

We have searched all the major databases for medical publications and the clinical trial registers. We did not identify any registered and completed clinical trial which has not reported the results over an extended period of time.

## 6 Discussion

### 6.1 Summary of main results

This systematic review included a total of eight RCTs. A total of 955 participants in seven RCTs were included in quantitative analysis. All comparisons other than that for ovarian cancer contained only one trial.

In people with gastric cancer and peritoneal metastases, there is very low certainty about the effect of CRS + HIPEC + systemic chemotherapy versus CRS + systemic chemotherapy or systemic chemotherapy.

In women with stage III or greater ovarian cancer undergoing interval CRS after chemotherapy, CRS + HIPEC + systemic chemotherapy probably results in improved survival compared to CRS + systemic chemotherapy.

In people with peritoneal metastases from colorectal cancer, the addition of HIPEC to CRS + systemic chemotherapy probably results in little to no difference in all-cause mortality or progression-free survival and results in increased complications compared to CRS + systemic chemotherapy. In the same patient group, the addition of CRS + HIPEC to systemic chemotherapy probably decreases all-cause mortality (compared to systemic chemotherapy alone).

The overall HRQoL was assessed only in ovarian cancer. CRS + HIPEC + systemic chemotherapy may result in little to no difference in overall HRQoL compared to CRS + systemic chemotherapy.

### 6.2 Controversies in interpretation of data

Clinical experts in treatment of peritoneal metastases have raised concerns about the PRODIGE-7 trial.^52^ We have discussed in detail the different concerns raised and why these concerns should not be used as a justification for not basing clinical practice on PRODIGE-7 trial in the full article. In summary, we based our clinical practice recommendations for colorectal peritoneal metastases on PRODIGE-7 trial because the trial was a low risk of bias trial for the comparison of HIPEC + CRS + systemic chemotherapy versus CRS + systemic chemotherapy, an appropriate analysis was used to analyze trial data, and there was no other trial of low of bias comparing HIPEC + CRS + systemic chemotherapy versus CRS + systemic chemotherapy. While the CRS + systemic chemotherapy was not directly compared with systemic chemotherapy alone, we recommended CRS + systemic chemotherapy in people with colorectal peritoneal metastases because of the lack of any “systemic chemotherapy alone” treatments that provide equivalent median survival as that observed in the control arm (CRS + systemic chemotherapy) in the PRODIGE-7 trial.

### 6.3 Certainty of evidence

The certainty of evidence was moderate for most comparisons. Most trials were at low risk of bias for all-cause mortality. Because of the nature of the comparison, it is not possible to blind the healthcare providers to the treatment groups. However, as per the RoB 2·0 tool, this does not result in bias because all-cause mortality is an objective outcome. The main reason for downgrading the evidence related to imprecision because of the small sample sizes in the trials and meta-analysis when relevant.

Overall, the balance of benefits and harms appear to be favorable for CRS + HIPEC + systemic chemotherapy versus CRS + systemic chemotherapy in ovarian cancer because of improvement in survival with CRS + HIPEC + systemic chemotherapy but not for other cancers. The balance of benefits and harms appear to be against the CRS + HIPEC + systemic chemotherapy versus CRS + systemic chemotherapy for colorectal cancer as the HIPEC group had more serious complications than CRS + systemic chemotherapy without an improvement in overall survival. Therefore, we have made strong recommendations for clinical practice for CRS + HIPEC + systemic chemotherapy versus CRS + systemic chemotherapy for ovarian cancers and against CRS + HIPEC + systemic chemotherapy for colorectal cancers.

### 6.4 Overall completeness and applicability of evidence

We included only gastric cancer, and ovarian cancer, colorectal cancer with peritoneal metastases. The participants included in the trials were adults who were likely to withstand major surgery. Most trials excluded people with extraperitoneal metastases. Therefore, these results are applicable in only people with metastases confined to the peritoneum.

It should be noted that all trials included in this review included systemic chemotherapy in both arms. Therefore, the evidence applies to people with peritoneal metastases receiving systemic chemotherapy.

The clinical recommendations related to CRS + systemic chemotherapy in colorectal peritoneal metastases are only applicable in centers with adequate expertize to select appropriate patients and perform CRS + systemic chemotherapy, as all the evidence supporting this treatment was from centers who were performing this (CRS + systemic chemotherapy) as part of CRS + HIPEC + systemic chemotherapy.

The results of this research and recommendations are applicable until the availability of the results of major new trials.

### 6.5 Potential biases in the review process

We performed a thorough search of literature. Two reviewers independently identified studies and extracted data. We followed the standard methodology for analyzing the data. These are the strengths of the review process.

We were unable to obtain IPD as planned. IPD would have allowed us to refine our effect estimates for subgroups of people with peritoneal metastases from colorectal, gastric, or ovarian cancer. It is difficult to estimate whether our conclusions would have changed if we had IPD; however, our systematic review and meta-analysis supports similar conclusions as the trial authors, suggesting that the impact of IPD may not be major enough to warrant an IPD once the health services have recovered from the impact of COVID-19.

### 6.6 Agreements and disagreements with other studies or reviews

This is the first systematic review on this topic. We agree with the individual study authors for all the comparisons.

For gastric cancer, we have indicated no recommendation as compared to the Italian Association of Medical oncology guidelines of strong recommendation against the use of CRS + HIPEC + systemic chemotherapy.^53^ Some potential reasons for the differences in recommendation may be differences in methodology. There were some differences in the estimation of hazard ratios of survival. However, even if we used the effect estimates used by methodologists involved in Italian Association of Medical oncology guidelines, our conclusions about uncertainty in evidence with gastric cancer would not have changed. The difference is likely to be due to the consideration of information from non-randomized studies in the recommendation by the Italian Association of Medical oncology guidelines. In practical terms though, in a state-funded healthcare system, our recommendations and those recommended by Italian Association of Medical oncology guidelines lead to the same result, that is, patients are not offered CRS + HIPEC + systemic chemotherapy routinely in clinical practice.

For colorectal cancers, we agree with the recent ESMO (European Society for Medical Oncology) Clinical Practice Guideline on metastatic colorectal cancer which suggested that HIPEC for colorectal peritoneal metastases should only be considered as part of well-designed clinical trials and CRS + systemic chemotherapy should be considered as the treatment of choice.^54^ We also agree with the recent ASCO (American Society of Clinical Oncology) guidelines on the treatment of metastatic colorectal cancer, which recommended against the routine clinical use (i.e., outside well-designed clinical trials) of CRS + HIPEC + systemic chemotherapy in people with colorectal peritoneal metastases.^55^

The ASCO guidelines provided a weak recommendation in favor of CRS + systemic chemotherapy for this group of patients while we have provided a strong recommendation in favor of CRS + systemic chemotherapy. The differences in the strength of recommendation is because of the following reason. Moderate certainty evidence indicated that CRS + HIPEC + systemic chemotherapy improved survival compared to systemic chemotherapy alone. While we acknowledge that the systemic chemotherapy used in the comparison of HIPEC + CRS + systemic chemotherapy is not the current treatment regimen used for disseminated colorectal cancers and the comparison was between HIPEC + CRS + systemic chemotherapy versus systemic chemotherapy alone (rather than CRS + systemic chemotherapy vs. systemic chemotherapy alone), the survival in the control arm of PRODIGE-7 suggests that using CRS + systemic chemotherapy can result in median survival of 41 months; the median survival of disseminated colorectal cancers in England between 2013 and 2017 was less than one year.^56^ This is indirect evidence for the survival benefit of CRS + systemic chemotherapy compared to systemic chemotherapy alone. However, because of the indirectness in evidence, the certainty of evidence will be downgraded to low. There are some situations that strong recommendations can be made using GRADE system despite low certainty evidence. As low certainty evidence suggests considerable survival benefit with CRS + systemic chemotherapy in a situation with very poor survival in the absence of CRS, we have made a strong recommendation for CRS + systemic chemotherapy when adequate expertize is available.

## 7 Conclusions

The role of CRS + HIPEC in gastric peritoneal metastases is uncertain. CRS + systemic chemotherapy should be standard of care for people with colorectal peritoneal metastases, with HIPEC given only as part of a randomized clinical trial focusing on subgroups and regimes. CRS + HIPEC should be standard of care in women with stage III or greater epithelial ovarian cancer undergoing interval CRS. Further well-designed RCTs are necessary.

## Supplementary Material

Appendix

## Figures and Tables

**Table 1:** Characteristics of included studies.

Study name	Type of primary cancer	Other major inclusion/exclusion criteria	Number randomized	Post-randomization exclusions	Mean or median age	Number of females (proportion)	Intervention versus control	Follow-up in months
Quénet 2021^50^	Colorectal cancer	Adults ≤70 years. Minor or moderate peritoneal carcinomatosis with a Sugarbaker peritoneal cancer index score ≤25. Macroscopically complete R1 surgical tumor reduction or of residual thickness not exceeding 1 mm (R2). Absence of extraperitoneal metastases (other than ovarian or retroperitoneal lymph node metastases).	265	0	60	133 (50·2%)	CRS + HIPEC (oxaliplatin-based) + systemic chemotherapy versus CRS +/− systemic chemotherapy	Median: 64
Verwaal 2003^51^	Colorectal cancer	Adults <71 years. No other distant metastases.	105	0	54	47 (44·8%)	CRS + HIPEC (mitomycin-based) + systemic chemotherapy versus systemic chemotherapy	Median: 22
Yang 2011^44^	Gastric cancer	Adults of 20–75 years. No other metastases other than to peritoneum.	68	0	50	33 (48·5%)	CRS + HIPEC (cisplatin + mitomycin-based) + systemic chemotherapy versus CRS +/− systemic chemotherapy	Median: 32
Rau 2021^45^	Gastric cancer	No other metastases other than to peritoneum or ovary. Possibility of 80% tumor reduction at cytoreductive surgery during diagnostic laparoscopy or exploratory laparotomy.	105	Not stated	Not stated	Not stated	CRS + HIPEC (cisplatin + mitomycin-based) + systemic chemotherapy versus CRS +/− systemic chemotherapy	Not stated
Rudloff 2014^46^	Gastric cancer	Potential for complete resection. No other metastases other than to peritoneum, liver, or lung.	17	0	48	7 (41·2%)	CRS + HIPEC (oxaliplatin-based) + systemic chemotherapy versus systemic chemotherapy	Minimum: 24
Van Driel 2018^49^	Ovarian cancer	Abdominal disease was too extensive for primary cytoreductive surgery or because surgery had been performed but was incomplete (i.e., after surgery, one or more residual tumors measuring >1 cm in diameter were present). No extra-abdominal metastases.	245	0	62	245 (100·0%)	CRS + HIPEC (cisplatin-based) + systemic chemotherapy versus CRS +/− systemic chemotherapy	Median: 57 months
Antonio 2022^47^	Ovarian cancer	No extraperitoneal metastases.	79	8 (unresectable)	61	79 (100·0%)	CRS + HIPEC (cisplatin-based) + systemic chemotherapy versus CRS +/ − systemic chemotherapy	Median: 32
Lim 2022^48^	Ovarian cancer	Adults <75 years. Residual tumors <1 cm Extraperitoneal metastases.	184	0	53	184 (100·0%)	CRS + HIPEC (cisplatin-based) + systemic chemotherapy versus CRS +/− systemic chemotherapy	Median: 70

Abbreviations: CRS, cytoreductive surgery; HIPEC, hyperthermic intraperitoneal chemotherapy.

**Table 2:** Risk of bias.

Study name	Bias arising from the randomization process	Bias due to deviations from intended interventions	Bias due to missing outcome data	Bias in measurement of the outcome	Bias in selection of the reported result	Overall risk of bias
Quénet 2021^50^	Low risk	Low risk	Low risk	Low risk	Some concerns	Low risk
Verwaal 2003^51^	Low risk	Low risk	Low risk	Low risk	Some concerns	Low risk
Yang 2011^44^	Some concerns	Low risk	Low risk	Low risk	Some concerns	Some concerns
Rudloff 2014^46^	Low risk	Low risk	Low risk	Low risk	Some concerns	Low risk
Rau 2021^45^	Some concerns	Some concerns	Low risk	Low risk	Some concerns	Some concerns
Antonio 2022^47^	Low risk	Low risk	Low risk	Low risk	Some concerns	Low risk
Van Driel 2018^49^	Low risk	Low risk	Low risk	Low risk	Low risk	Low risk
Lim 2022^48^	Low risk	Low risk	Low risk	Low risk	Some concerns	Low risk

**Table 3:** Certainty of evidence.

Outcomes	Anticipated absolute effects* (95% CI)			Relative effect (95% CI)	No of participants (studies)	Certainty of the evidence (GRADE)	Comments
	Risk with CRS		Risk with CRS + HIPEC				
Colorectal cancer: CRS + HIPEC + systemic chemotherapy versus CRS + systemic chemotherapy							
All-cause mortality (median follow-up: 64 months)	606 per 1000		606 per 1000 (444–771)	HR 1·00 (0·63–1·58)	265 (1 RCT)	⊕⊕⊕◯ Moderate^a^	
Serious adverse events (short-term)	152 per 1000		256 per 1000 (156–420)	RR 1·69 (1·03–2·77)	265 (1 RCT)	⊕⊕◯◯ Low^a^^,^^b^	
Time to disease progression (median follow-up: 64 months)	841 per 1000		812 per 1000 (734–881)	HR 0·91 (0·72–1·16)	265 (1 RCT)	⊕⊕◯◯ Low^a^^,^^b^	
Colorectal cancer: CRS + HIPEC + systemic chemotherapy versus systemic chemotherapy alone							
All-cause mortality (median follow-up: 22 months)	608 per 1000		402 per 1000 (259–589)	HR 0·55 (0·32–0·95)	105 (1 RCT)	⊕⊕⊕◯ Moderate^a^	
Gastric cancer: CRS + HIPEC + systemic chemotherapy versus CRS + systemic chemotherapy							
All-cause mortality (median follow-up 32 months)	971 per 1000		738 per 1000 (523–915)	HR 0·38 (0·21–0·70)	68 (1 RCT)	⊕◯◯◯ very low^a^’^c^’^d^	Another trial including 105 participants indicated that there was no difference in all-cause mortality between the two groups but could not be included in the analysis because the numbers were not reported in a format suitable for analysis
Serious adverse events (short-term)	118 per 1000		147 per 1000 (44–501)	RR 1·25 (0·37–4·26)	68 (1 RCT)	⊕◯◯◯ very low^a,b,c^	
Gastric cancer: CRS + HIPEC + systemic chemotherapy versus systemic chemotherapy alone							
All-cause mortality (minimum follow-up 24 months)	1000 per 1000	1000 per 1000 (1000–1000)		HR 0·40 (0·30–0·52)	17 (1 RCT)	⊕⊕⊕◯ Moderate^a^	
Ovarian cancer: CRS + HIPEC + systemic chemotherapy versus CRS + systemic chemotherapy							
All-cause mortality (median follow-up: 32–70 months)	574 per 1000	463 per 1000 (385–547)		HR 0·73 (0·57–0·93)	500 (3 RCTs)	⊕⊕⊕◯ Moderate^a^	
Health-related quality of life assessed with: Global health status	The mean health-related quality of life was 69.79	MD 4.85 more (7.74 fewer to 17.44 more)		-	71 (1 RCT)	⊕⊕◯◯ Low^a,b^	
Scale from: 0–100							
Mean follow-up: 12 months							
Serious adverse events (proportion) (short-term)	252 per 1000	267 per 1000 (184–387)		RR 1·06 (0·73–1·54)	316 (2 RCTs)	⊕⊕◯◯ Low^a,b^	
Serious adverse events (number per participant) (short-term)	326 per 1000	414 per 1000 (355–486)		Rate ratio 1·27 (1·09–1·49)	184 (1 RCT)	⊕⊕⊕◯ Moderate^b^	
Time to disease progression (median follow-up: 32–70 months)	857 per 1000	758 per 1000 (688–822)		HR 0·73 (0·60–0·89)	500 (3 RCTs)	⊕⊕◯◯ Low^a,b^	

*Note*: Explanations.

Abbreviations: assessment, development and evaluations; CRS, cytoreductive surgery; GRADE, grading of recommendations; HIPEC, hyperthermic intraperitoneal chemotherapy.

a Downgraded one level for imprecision.

b Downgraded one level for lack of blinding for a subjective outcome.

c Downgraded one level for unclear randomization.

d Downgraded one level for heterogeneity in the results between the study that reported data in analyzable format compared to the trial that did not report data in analyzable format.

## Data Availability

The data collected for the study will be available as tables in the study and the appendix. The data includes information extracted from the study to calculate HR, risk ratio (RR), rate ratio, or MD with 95% confidence intervals (95% CI). We have also included the information on ongoing studies. We did not obtain IPD.
