# Supplementary material for: Cytoreductive surgery plus hyperthermic intraoperative peritoneal chemotherapy for people with peritoneal metastases from colorectal, ovarian or gastric origin: A systematic review of randomized controlled trials
Source: World J Surg. Author manuscript; Available in PMC 2024 Dec 9. (PMC7617159; doi:10.1002/wjs.12186)
Supplement: Appendix [file EMS197885-supplement-Appendix.pdf]

SUPPORTING INFORMATION

Additional supporting information can be found online in the Supporting Information section at the end of this article.

APPENDIX A: SEARCH STRATEGIES

Medline

1. Hyperthermia, Induced/
2. ((hyperthermic or heated) adj3 (intraperitoneal or intra-peritoneal) adj3 (chemotherapy or chemotherapies)).ti,ab.
3. (intraperitoneal adj3 chemohyperthermia).ti,ab.
4. (HIPEC or IPHC or HIIC).ti,ab.
5. 1 or 2 or 3 or 4
6. Cytoreduction Surgical Procedures/
7. ((cytoreductive or cytoreduction or debulking) adj3 (surgery or surgeries or surgical or procedure or procedures)).ti,ab.
8. 6 or 7
9. 5 or 8
10. exp Colorectal Neoplasms/
11. exp Ovarian Neoplasms/
12. Stomach Neoplasms/
13. ((colorectal or bowel or colon or colonic or rectum or rectal or ovary or ovaries or ovarian or gastric or stomach) adj3 (cancer or cancers or carcinoma or carcinomas or tumor or tumors or tumor or tumors or neoplasm or neoplasms)).ti,ab.
14. 10 or 11 or 12 or 13
15. 9 and 14
16. randomised controlled trial.pt.
17. Controlled clinical trial.pt.
18. randomised.ab.
19. placebo.ab.
20. drug therapy.fs.
21. randomly.ab.
22. trial.ab.
23. groups.ab.
24. 16 or 17 or 18 or 19 or 20 or 21 or 22 or 23
25. exp animals/not humans.sh.
26. 24 not 25
27. 15 and 26
28. (cost: or cost benefit analys: or health care costs).mp.
29. 15 and 28
30. 27 or 29

Embase

1. hyperthermic intraperitoneal chemotherapy/
2. ((hyperthermic or heated) adj3 (intraperitoneal or intra-peritoneal) adj3 (chemotherapy or chemotherapies)).ti,ab.
3. (intraperitoneal adj3 chemohyperthermia).ti,ab.
4. (HIPEC or IPHC or HIIC).ti,ab.
5. 1 or 2 or 3 or 4
6. cytoreductive surgery/
7. ((cytoreductive or cytoreduction or debulking) adj3 (surgery or surgeries or surgical or procedure or procedures)).ti,ab.
8. 6 or 7
9. 5 or 8
10. exp colon cancer/
11. exp rectum cancer/
12. exp ovary cancer/
13. exp stomach cancer/
14. ((colorectal or bowel or colon or colonic or rectum or rectal or ovary or ovaries or ovarian or gastric or stomach) adj3 (cancer or cancers or carcinoma or carcinomas or tumor or tumors or tumor or tumors or neoplasm or neoplasms)).ti,ab.
15. 10 or 11 or 12 or 13 or 14
16. 9 and 15
17. exp crossover-procedure/or exp double-blind procedure/or exp randomised controlled trial/or single-blind procedure/
18. (((((random* or factorial* or crossover* or cross over* or cross-over* or placebo* or double*) adj blind*) or single*) adj blind*) or assign* or ervice* or volunteer*).af.
19. 17 or 18
20. 16 and 19
21. (cost or costs).tw.
22. 16 and 21
23. 20 or 22

Cochrane

1. MeSH descriptor: [Hyperthermia, Induced] this term only
2. ((hyperthermic or heated) near/3 (intraperitoneal or intra-peritoneal) near/3 (chemotherapy or chemotherapies))
3. (intraperitoneal near/3 chemohyperthermia)
4. (HIPEC or IPHC or HIIC)
5. #1 or #2 or #3 or #4
6. MeSH descriptor: [Cytoreduction Surgical Procedures] this term only
7. ((cytoreductive or cytoreduction or debulking) near/3 (surgery or surgeries or surgical or procedure or procedures))
8. #6 or #7
9. #5 or #8
10. MeSH descriptor: [Colorectal Neoplasms] explode all trees
11. MeSH descriptor: [Ovarian Neoplasms] explode all trees
12. MeSH descriptor: [Stomach Neoplasms] this term only
13. ((colorectal or bowel or colon or colonic or rectum or rectal or ovary or ovaries or ovarian or gastric or

14322323, 2024, 6, Downloaded from https://onlinelibrary.wiley.com/doi/10.1002/wjs.12186 by University College London UCL Library Services, Wiley Online Library on [31/07/2024]. See the Terms and Conditions (https://onlinelibrary.wiley.com/terms-and-conditions) on Wiley Online Library for rules of use; OA articles are governed by the applicable Creative Commons License

stomach) near/3 (cancer or cancers or carcinoma or carcinomas or tumor or tumors or tumor or tumors or neoplasm or neoplasms))
14. #10 or #11 or #12 or #13
15. #9 and #14

Science citation index

1. TS=((hyperthermic or heated) near/3 (intraperitoneal or intra-peritoneal) near/3 (chemotherapy or chemotherapies))
2. TS=(intraperitoneal near/3 chemohyperthermia)
3. TS=(HIPEC or IPHC or HIIC)
4. #3 OR #2 OR #1
5. TS=((cytoreductive or cytoreduction or debulking) near/3 (surgery or surger-ies or surgical or procedure or procedures))
6. #5 or #4
7. TS=((colorectal or bowel or colon or colonic or rectum or rectal or ovary or ovaries or ovarian or gastric or stomach) near/3 (cancer or cancers or carci-noma or carcinomas or tumor or tumors or tumor or tumors or neoplasm or neoplasms))
8. TS=(random* or placebo* or blind* or meta-analysis or cost or costs)
9. #8 AND #7 AND #6

WHO trials register

Condition: colorectal OR bowel OR colon OR colonic OR rectum OR rectal OR ovary OR ovaries OR ovarian OR gastric OR stomach.

Intervention: HIPEC OR hyperthermic intraperitoneal chemotherapy OR IPHC OR intraperitoneal chemohyperthermia OR HIIC OR heated intraoperative intraperitoneal chemotherapy OR cytoreductive surgery OR CRS.

ClinicalTrials.gov

Condition: colorectal OR bowel OR colon OR colonic OR rectum OR rectal OR ovary OR ovaries OR ovarian OR gastric OR stomach.

Study Type: Interventional Studies (Clinical Trials).

Intervention/treatment: HIPEC OR hyperthermic intraperitoneal chemotherapy OR IPHC OR intraperitoneal chemohyperthermia OR HIIC OR heated intraoperative intraperitoneal chemotherapy OR CRS OR CRS.

Interventional studies, phase 2,3,4.

Interventional Studies | colorectal OR bowel OR colon OR colonic OR rectum OR rectal OR ovary OR ovaries OR ovarian OR gastric OR stomach | HIPEC OR hyperthermic intraperitoneal chemotherapy OR IPHC OR intraperitoneal chemohyperthermia OR HIIC OR heated intraoperative intraperitoneal chemotherapy OR CRS OR CRS | Phase 2, 3, 4

Cost-effectiveness analysis (CEA) registry

The following terms were searched:

Hyperthermic
Cytoreduction
Cytoreductive

APPENDIX B

TABLE B1 Details of hyperthermic intraoperative peritoneal chemotherapy and systemic chemotherapy received.

Study name	Type of primary cancer	HIPEC	Systemic chemotherapy	Was systemic chemotherapy given pre-operatively
Quénet 2021[50]	Colorectal cancer	HIPEC was administered with either the closed or open abdomen techniques according to each center's standard approach. In both approaches, systemic chemotherapy (400 mg/m^2 fluorouracil and 20 mg/m^2 folinic acid) was delivered intravenously 20 min before intraperitoneal infusion of oxaliplatin (460 mg/m^2 if the open technique was used and 360 mg/m^2 if the closed technique was used) in 2 L/m^2 of dextrose at 43°C over 30 min.	The chemotherapy and targeted therapy regimens used were at investigators' discretion. 110 patients in cytoreductive surgery plus HIPEC group and 109 in the cytoreductive surgery alone group were treated with preoperative chemotherapy. Patients in both groups received a median of six cycles of preoperative chemotherapy. 48 (44%) of 133 patients in the HIPEC group and 46 (42%) of patients in the surgery only group received preoperative oxaliplatin-based treatment.	219/265 (82·6%) received pre-operative chemotherapy

(Continues)

14322323, 2024, 6, Downloaded from https://onlinelibrary.wiley.com/doi/10.1002/wjs.12186 by University College London UCL Library Services, Wiley Online Library on [31/07/2024]. See the Terms and Conditions (https://onlinelibrary.wiley.com/terms-and-conditions) on Wiley Online Library for rules of use; OA articles are governed by the applicable Creative Commons License

TABLE B1 (Continued)

Study name	Type of primary cancer	HIPEC	Systemic chemotherapy	Was systemic chemotherapy given pre-operatively
Verwaal 2003[51]	Colorectal cancer	To increase the volume of the abdominal cavity and to prevent spillage of lavage fluid, the skin of the laparotomy wound was pulled up against a retractor. A plastic sheet covered the laparotomy opening to reduce heat loss and to avoid drug spilling. A central aperture was made to allow manipulation to achieve optimal drug and heat distribution. The perfusion circuit consisted of a centrally placed inflow catheter, outflow catheters, placement in the pelvis below left and right diaphragm, a roller pump, and a heat exchanger. Temperature probes were attached to inflow and outflow catheters. Perfusion was started with a minimum of 3 L of isotonic dialysis fluid, at 1–2 L/min, and an inflow temperature of 41°C–42°C. As soon as the temperature in the abdomen was stable above 40°C, MMC (mitomycin) was added to the perfusate at a dose of 17·5 mg/m^2 followed by 8·8 mg/m^2 every 30 min. The total dose was limited to 70 mg at maximum. If the core temperature exceeded 39°C, the inflow temperature was reduced. After 90 min, the perfusion fluid was drained from the abdomen, and bowel continuity was restored.	Chemotherapy was given in the local setting, usually by the patients' own medical oncologist, and consisted of fluorouracil (intravenous [IV] push-dose of 400 mg/m^2) and leucovorin (IV 80 mg/m^2) on an outpatient basis (modified Laufman regimen). Treatment was given weekly for 26 weeks, or until progression, death, or unacceptable toxicity.	No
Yang 2011[44]	Gastric cancer	After surgery, HIPEC was performed before closure of abdominal cavity, as this open technique is believed to provide optimal thermal homogeneity and spatial diffusion, with 120 mg of cisplatin and 30 mg of mitomycin C each dissolved 6 l of heated saline (drug concentration cisplatin 20 lg/mL, mitomycin C 5 lg/mL). An outflow tube for perfusion was placed in Douglas' pouch just before HIPEC. The heated perfusion solution was infused into the peritoneal cavity at a rate of 500 mL/min through the inflow tube introduced from an automatic hyperthermia chemotherapy perfusion device (ES-6001, Wuhan E-sea Digital engineering, Wuhan, China). The skin of the abdomen is attached to a retractor ring and a plastic sheet covered the open wound to keep the temperature stable. The perfusion in the peritoneal cavity was stirred manually with care not to infuse directly on the bowel surface. The temperature of the perfusion solution in peritoneal space was kept at 43·0 ± 0·5°C and monitored with a thermometer on real time. The total HIPEC time was 60–	Not stated	Not stated

14322323, 2024, 6, Downloaded from https://onlinelibrary.wiley.com/doi/10.1002/wjs.12186 by University College London UCL Library Services, Wiley Online Library on [31/07/2024]. See the Terms and Conditions (https://onlinelibrary.wiley.com/terms-and-conditions) on Wiley Online Library for rules of use; OA articles are governed by the applicable Creative Commons License

TABLE B1 (Continued)

Study name	Type of primary cancer	HIPEC	Systemic chemotherapy	Was systemic chemotherapy given pre-operatively
		90 min, after which the perfusion solution in the abdominal cavity was removed through the suction tube, and drainage tubes were placed at appropriate sites depending on the type of primary operation.		
Rau 2021[45]	Gastric cancer	CRS + The HIPEC treatment consisted of mitomycin C 15 mg/m^2 and Cisplatin 75 mg/m^2, in 5 L of saline (60 min, 42°C)	Preoperative chemotherapy 3 cycles, each cycle 21 days. Patients with negative or unknown HER-2 status receive epirubicin 50 mg/m^2 infusion (maximum 100 mg/d). Oxaliplatin 130 mg/m^2 infusion (maximum 260 mg/d) and capecitabine oral 625 mg/m^2 two times a day (maximum 2500 mg/d).<br>Patients with positive HER-2 status received.<br>Cisplatin: 80 mg/m^2 infusion (maximum of 160 mg/d). Capecitabine: Oral 1000 mg/m^2 (two times a day maximum of 4000 mg/d), on day 1–14.<br>Trastuzumab: 8 mg/kg infusion (on cycle 1 and 6 mg/kg on cycle 2 and 3).<br>4–12 weeks after surgery, 3 cycles of postoperative chemotherapy were applied.	Yes
Rudloff 2014[46]	Gastric cancer	Hyperthermic intraperitoneal chemotherapy (HIPEC) was administered using a closed circuit of oxaliplatin solution at 460 mg/m^2 in 5% dextrose in water (D5W) at 41°C for 30 min. Prior to perfusion a single dose each of fluorouracil (5-FU) 400 mg/m^2 IV in 50 mL D5W and leucovorin 20 mg/m^2 IV in 50 mL D5W were administered over 5 min to enhance the effect of regional oxaliplatin delivered IP. The perfusion flow rate was then maintained at ~2·0 L/min and a perfusate volume, which moderately distends the abdominal cavity, correlating with intraabdominal pressures of 5–15 mm Hg (2·0 L/m^2).	Within 14 days of study randomization patients began FOLFIXIRI treatment (in the systemic chemotherapy arm; in the CRS + HIPEC arm, systemic chemotherapy was started within 8 weeks of surgical resection). Systemic chemotherapy was administered once every 14 days, and repeated for 12 cycles (approximately 6 months). On treatment day #1 irinotecan was administered IV over 90 min followed by leucovorin and oxaliplatin, given concomitantly over 2 h, followed by 5-FU given via continuous infusion (CIV) over 48 h.	No
Van Driel 2018[49]	Ovarian cancer	HIPEC was administered at the end of the cytoreductive surgical procedure with the use of the open technique. In brief, the abdomen was filled with saline that circulated continuously with the use of a roller pump through a heat exchanger. By circulation of the heated saline, an intraabdominal temperature of 40°C (104°F) was maintained. Perfusion with cisplatin at a dose of 100 mg per square meter and at a flow rate of 1 L per minute was then initiated (with 50% of the dose perfused initially,	Patients received three cycles of neoadjuvant chemotherapy with carboplatin (area under the curve of 5–6 mg per milliliter per minute) and paclitaxel (175 mg per square meter of body-surface area). Patients received an additional three cycles of carboplatin and paclitaxel after surgery.	Yes

(Continues)

14322323, 2024, 6, Downloaded from https://onlinelibrary.wiley.com/doi/10.1002/wjs.12186 by University College London UCL Library Services, Wiley Online Library on [31/07/2024]. See the Terms and Conditions (https://onlinelibrary.wiley.com/terms-and-conditions) on Wiley Online Library for rules of use; OA articles are governed by the applicable Creative Commons License

TABLE B1 (Continued)

Study name	Type of primary cancer	HIPEC	Systemic chemotherapy	Was systemic chemotherapy given pre-operatively
		25% at 30 min, and 25% at 60 min). The perfusion volume was adjusted such that the entire abdomen was exposed to the perfusate. The HIPEC procedure took 120 min in total, including the 90-min perfusion period. At the end of the perfusion, drains were used to empty the abdominal cavity as completely as possible. To prevent nephrotoxicity, sodium thiosulphate was administered at the start of perfusion as an intravenous bolus (9 g per square meter in 200 mL), followed by a continuous infusion (12 g per square meter in 1000 mL) over 6 h.		
Antonio 2022[47]	Ovarian cancer	At the end of the surgery, HIPEC was administered by the open technique (Coliseum) to the patients of the experimental arm according to the following scheme: Cisplatin 75 mg/m^2 diluted for perfusion in 3 L of dialysis fluid (Dialisan, Shanghai Plop medical technology Co., Ltd. China), with circulation maintained in a constant flow of 0·5 to 0·7 L/min longer than 60 min. Two intra-abdominal thermometers positioned in the pelvis and diaphragmatic area were used to monitor the temperature during perfusion, with maintenance of a constant temperature between 42 and 43·8°C. During the intervention, the temperature was strictly controlled through an esophageal thermometer, with the objective of keeping the patient normothermic (37·8°C), using physical measures and serotherapy	All the patients were treated with a minimum of three cycles of systemic NACT with carboplatin (AUC 5) and paclitaxel (175 mg/m^2) before surgery. After recovery and hospital discharge, up to six cycles of systemic adjuvant chemotherapy were completed per patient with the same carboplatin and paclitaxel scheme.	Yes
Lim 2022[48]	Ovarian cancer	Intraoperative HIPEC (75 mg/m^2 of cisplatin) was perfused through a closed technique with a target temperature of 41·5°C for 90 min using the Belmont hyperthermia pump system (Belmont instrument Corporation), women randomized to the HIPEC group received blanket cooling, intravenous cold fluid hydration, and ice pack application over the head before and during HIPEC procedures. After the cytoreductive and reconstructive surgical procedures, 2 inflow and 2 outflow tubes were placed in the pelvic cavity and in the subdiaphragmatic space, respectively. The abdominal wall was closed in layers with a water-tight fit, and 0.9% normal saline was injected into the closed abdominal cavity. After smooth circulation to and from the HIPEC pump was confirmed,	During postoperative recovery, if the patients could tolerate a general diet without evidence of active infection and with an acceptable clinical condition to sustain chemotherapy, we administered 6 cycles of intravenous paclitaxel and carboplatin in both groups.	77/184 (41·8%) received pre-operative chemotherapy

14322323, 2024, 6, Downloaded from https://onlinelibrary.wiley.com/doi/10.1002/wjs.12186 by University College London UCL Library Services, Wiley Online Library on [31/07/2024]. See the Terms and Conditions (https://onlinelibrary.wiley.com/terms-and-conditions) on Wiley Online Library for rules of use; OA articles are governed by the applicable Creative Commons License

TABLE B1 (Continued)

Study name	Type of primary cancer	HIPEC	Systemic chemotherapy	Was systemic chemotherapy given pre-operatively
		the chemotherapeutic agent was mixed with the circulating fluid. During the 90-min HIPEC perfusion procedure, the patients were gently shaken from side to side to ensure even distribution of the chemotherapeutic agent within the peritoneal cavity. Sodium thiosulfate was not used in the initial 71 cases, given the low incidence of serum creatinine elevation in the phase 2 study. However, in the remaining 21 patients, 4 g/m2 of sodium thiosulfate was administered as a bolus infusion immediately before HIPEC, and 12 g/m2 was administered over 6 h during and after the HIPEC procedures.		

Abbreviations: CRS, cytoreductive surgery; HIPEC, hyperthermic intraperitoneal chemotherapy; NACT, Neoadjuvant chemotherapy.

TABLE B2 Summary of hyperthermic intraoperative peritoneal chemotherapy performed.

Study name	Type of primary cancer	Drugs	Temperature (centigrade)	Duration	Technique (open or closed)
Quénet 2021[50]	Colorectal cancer	Oxaliplatin (IP) + IV fluorouracil + IV folinic acid	43°	30 min	Either
Verwaal 2003[51]	Colorectal cancer	Mitomycin	41–42°	90 min	Open
Yang 2011[44]	Gastric cancer	Cisplatin + mitomycin	43°	60–90 min	Open
Rau 2021[45]	Gastric cancer	Cisplatin + mitomycin	42°	60 min	Not stated
Rudloff 2014[46]	Gastric cancer	Oxaliplatin (IP) + IV fluorouracil + IV folinic acid	41°	30 min	Closed
Van Driel 2018[49]	Ovarian cancer	Cisplatin	40°	90 min	Open
Antonio 2022[47]	Ovarian cancer	Cisplatin	42–43•8°	60 min	Open
Lim 2022[48]	Ovarian cancer	Cisplatin	41•5°	90 min	Closed

14322323, 2024, 6, Downloaded from https://onlinelibrary.wiley.com/doi/10.1002/wjs.12186 by University College London UCL Library Services, Wiley Online Library on [31/07/2024]. See the Terms and Conditions (https://onlinelibrary.wiley.com/terms-and-conditions) on Wiley Online Library for rules of use; OA articles are governed by the applicable Creative Commons License
